# Stress-Related Roles of Exosomes and Exosomal miRNAs in Common Neuropsychiatric Disorders

**DOI:** 10.3390/ijms25158256

**Published:** 2024-07-29

**Authors:** Myrsini Chamakioti, George P. Chrousos, Eva Kassi, Dimitrios Vlachakis, Christos Yapijakis

**Affiliations:** 1 Unit of Orofacial Genetics, 1st Department of Pediatrics, National Kapodistrian University of Athens, “Aghia Sophia” Children’s Hospital, 115 27 Athens, Greece; m.chamakioti@gmail.com; 2 University Research Institute of Maternal and Child Health and Precision Medicine, Choremion Laboratory, “Aghia Sophia” Children’s Hospital, 115 27 Athens, Greece; chrousge@med.uoa.gr; 3 1st Department of Internal Medicine, School of Medicine, National Kapodistrian University of Athens, Laikon Hospital, 115 27 Athens, Greece; ekassi@med.uoa.gr; 4 Laboratory of Genetics, Department of Biotechnology, School of Applied Biology and Biotechnology, Agricultural University of Athens, 118 55 Athens, Greece; dimvl@aua.gr

**Keywords:** exosomes, microRNAs, stress response, synaptic plasticity, neurogenesis, major depression, bipolar disorder, schizophrenia, Alzheimer’s disease, Huntington’s disease

## Abstract

Exosomes, natural nanovesicles that contain a cargo of biologically active molecules such as lipids, proteins, and nucleic acids, are released from cells to the extracellular environment. They then act as autocrine, paracrine, or endocrine mediators of communication between cells by delivering their cargo into recipient cells and causing downstream effects. Exosomes are greatly enriched in miRNAs, which are small non-coding RNAs that act both as cytoplasmic post-transcriptional repression agents, modulating the translation of mRNAs into proteins, as well as nuclear transcriptional gene activators. Neuronal exosomal miRNAs have important physiologic functions in the central nervous system (CNS), including cell-to-cell communication, synaptic plasticity, and neurogenesis, as well as modulating stress and inflammatory responses. Stress-induced changes in exosomal functions include effects on neurogenesis and neuroinflammation, which can lead to the appearance of various neuropsychiatric disorders such as schizophrenia, major depression, bipolar disorder, and Alzheimer’s and Huntington’s diseases. The current knowledge regarding the roles of exosomes in the pathophysiology of common mental disorders is discussed in this review.

## 1. Introduction

### 1.1. Exosomes and Their Functions

As part of their physiological functions, virtually all cells release nanovesicles known as exosomes. These belong to the broader category of extracellular vesicles (EVs), a group of vesicles surrounded by a lipid bilayer membrane and released from cells to the extracellular environment. Apart from exosomes (40–200 nm), other types of EVs are microvesicles (950–1000 nm) and apoptotic bodies (500–2000 nm) [1].

Exosomes contain a cargo of biologically active molecules such as lipids, proteins, and nucleic acids. Their main role is to act as autocrine, paracrine, or endocrine mediators of communication between cells [2,3]. Another important role of exosomes is to discard lysosomal materials from the cell such as misfolded proteins or cytoplasmic DNA [4]. Exosomes have crucial roles in a wide variety of biological processes, namely the stress and immune responses, as well as cell differentiation, angiogenesis, and tissue regeneration. Last but not least, exosomes are part of the pathophysiologic mechanisms of various diseases such as heart failure, cancer, and neurodegenerative disorders [5].

### 1.2. The Biogenesis of Exosomes

**Figure 1:**
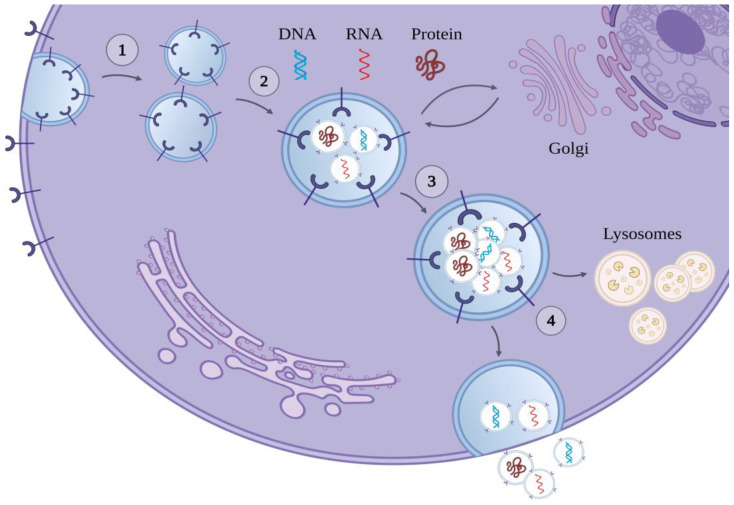
Biogenesis of exosomes: (1) the cell membrane invaginates and forms the early endosome; (2) the early endosome is loaded with its cargo, which can be DNA, RNA, or proteins; (3) the multivesicular body is formed; (4) and the multivesicular body is either degraded by lysosomes or fuses with the cell membrane and releases its intracavitary vesicles in the extracellular space. Once released, the intracavitary vesicles are named exosomes.

The process by which exosomes are manufactured starts inside the cell, within the endosomes. The constant folding of the endosomal envelope gives rise to a large number of intraluminal vesicles (ILVs). The resultant mature endosomes, called multivesicular bodies (MVBs), act by transferring molecules marked by mono-ubiquitination or by tetraspanins. The endosomal sorting complex required for transport (ESCRT), a multi-subunit, cytoplasmic protein machinery that takes part in membrane budding and tearing [6], coordinates the ESCRT-dependent pathway, where the mono-ubiquitinated proteins are assembled into the vesicles. In the ESCRT-independent pathway, where tetraspanins and the lipid ceramide play a crucial role, the incorporation of the cargo into the vesicles and the configuration of the membrane curvature is controlled [7,8]. Following their assembly, the MVBs utilize the cell cytoskeleton to carry themselves to the plasma membrane and fuse with it (Figure 1). The fusion of the MVBs with the plasma membrane is regulated by the cell-specific GTPase family of Rab proteins and the SNARE protein complex (SNAP receptor proteins) [9,10].

By the time the ILVs are released by the MVBs into the extracellular space, they are called exosomes. The exosomes then can be identified by protein biomarkers such as the previously mentioned tetraspanins (CD9, CD63, CD81, CD82), the ALG2-interacting protein X (Alix), the heat shock protein 70 (Hsp70), and the tumor susceptibility gene 101 (TSG101) protein [11,12]. The specific composition of exosomes is affected by a variety of factors, such as the cell origin and metabolic status, the cell release site, and the physio-logic context of the cell [13]. Exosomes may be transported through biological fluids, such as plasma, to deliver their cargo into recipient cells and to cause downstream effects, including the triggering of signaling cascades [10,14].

Exosomes may interact with and transfer their cargo to their target cells through cell surface adhesion molecules (phosphatidylserine receptors, lectins, integrins, etc.) in one of the following ways:

1. Exosomal membrane proteins directly activate membrane receptors of target cells without being internalized;
2. Exosomal membrane proteins are cleaved by proteases and the resultant soluble fragments bind to membrane receptors of the target cells;
3. Exosomes are internalized into the target cells via endocytosis, phagocytosis, or direct fusion with the plasma membranes of the target cells.

### 1.3. The Cargo of Exosomes

As mentioned above, exosomes contain a complex cargo of proteins, lipids, and nucleic acids [2]:

(a) Their protein cargo consists of the most commonly expressed endosomal proteins incorporated during the biogenesis of the MVBs (Alix, TSG101, heat shock proteins Hsc/Hsp 70 and 90), the vesicular transport and fusion proteins (Rab GTPases, SNAREs, annexins, and flotillin), and the proteins that interact with the target cell integrins and tetraspanins. Apart from the above, however, the protein composition of each exosome is diverse and depends on the cell type of origin and its physiologic or pathologic state [11].

(b) Their lipid cargo includes cholesterol, sphingolipids (e.g., ceramide), and glycerophospholipids [15].

(c) Their nucleic acid cargo includes DNA, mRNA, and non-coding RNAs (ncRNAs). The latter category is subdivided into long non-coding RNAs (lncRNAs) and short ncRNAs—including microRNAs (miRNAs)—that react with other nucleic acids and proteins of the target cell and dynamically modify its gene expression and protein translation.

### 1.4. Exosomal miRNAs: Biogenesis and Function as Modulators of Gene Expression

Interestingly, in comparison to other biological fluids, exosomes are greatly enriched in miRNAs [16]. MicroRNAs are short ncRNAs with an average length of 19–24 nucleotides [17,18]. They comprise one of the most substantial subcategories of ncRNAs and are carried throughout the body, mainly as exosomal cargos [19,20]. miRNAs may vary significantly in terms of nucleotide sequence. However, they all function as post-transcriptional modification agents. This regulatory function of microRNAs is accomplished through the RNA-induced silencing complex (RISC), which comprises a miRNA molecule and an AGO protein. By recognizing and binding to its mRNA targets through Watson and Crick base pairing, miRNA targets the mRNA to be modified. More specifically, the seed sequence of the miRNA (situated at positions 2–8 at the 5′ end) binds to its responsive element, which is most commonly located at the 3′ untranslated region (UTR) of the target mRNA [21,22]. The grade and nature of the complementarity between the microRNA and target, which is cell- and tissue-specific throughout the body, determines the mechanism of gene modification [23]. Next, given that the miRNA–mRNA base pairing is perfectly complementary, AGO2 acts as an endonuclease and cleaves the target mRNA [24,25].

**Figure 2:**
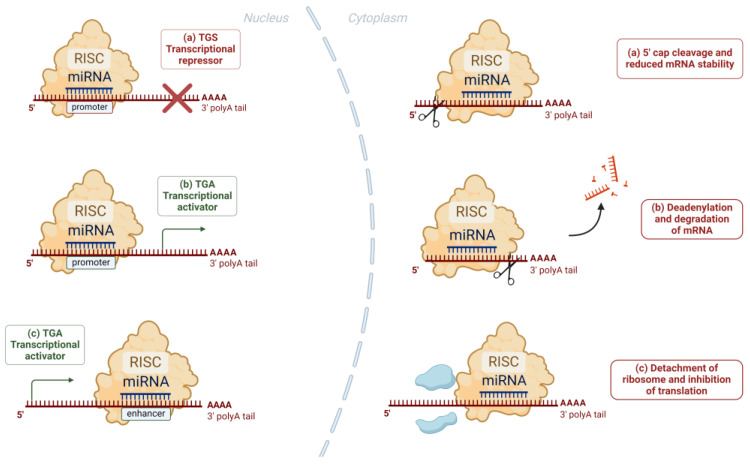
Nucleic and cytoplasmic actions of miRNAs: In the nucleus, the miRNA–RISC complex binds with complementary sequences of promoters or enhancers and causes transcriptional repression (**a**) or activation (**b**,**c**). In the cytoplasm, the binding of the miRNA–RISC complex to the target mRNA can result in (**a**) cleavage of the 5′ cap of the target mRNA and reduced stability, (**b**) deadenylation and subsequent degradation of the target mRNA, and (**c**) detachment of the ribosome and inhibition of the translation process.

Apart from the canonical cytoplasmic action of miRNAs, recent studies have revealed the much rarer action of miRNAs in the nucleus of the cell. Through the formation of complexes that interact with gene promoter and enhancer regions and through synergistic interaction with other miRNAs, miRNAs up- or down-regulate gene transcription (Figure 2) [26].

**Figure 3:**
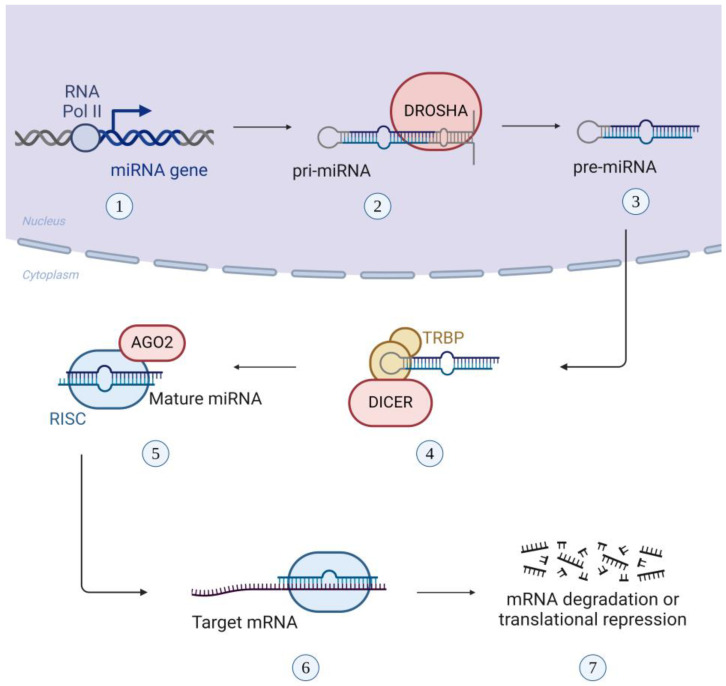
Biogenesis of miRNA: (1) RNA polymerase II transcribes the miRNA gene into pri-miRNA; (2) RNase III endonuclease, Drosha, cleaves the pri-miRNA, creating the pre-miRNA; (3) pre-miRNA is exported to the cytoplasm; (4) the cytoplasmic RNase III enzyme, Dicer, and the TAR RNA binding protein (TRBP) convert the pre-miRNA into double-stranded miRNA; (5) the mature miRNA binds to RNA-induced silencing complex (RISC) and then gets unchained by the Argonaute 2 protein (AGO2); (6) the mature miRNA remains bound to RISC and binds complementary with one strand to the target mRNA while the other strand is hydrolyzed; and (7) the interaction of the miRNA with the mRNA results in mRNA degradation or translational repression.

The biogenesis of miRNAs begins in the nucleus, where RNA polymerases II and III transcribe the corresponding genes into several-kilobase-long, hairpin-loop pri-miRNAs [27]. Following this, pri-miRNAs are cut up by the enzymatic complex Drosha-DiGeorge syndrome Critical Region gene 8 (Drosha-DGCR8) into stem-loop pre-miRNAs [18,28]. The pre-miRNAs are transferred to the cytoplasm by exportin 5 and Ran GTPase, where they are processed by the RNAse III enzyme Dicer and the transactivation-responsive RNA binding protein (TRBP). These enzymes produce a mature, about 22nt-long, double-stranded miRNA with a 5′ phosphate end [29]. The double-stranded miRNA becomes unwinded, producing two strands. One strand is degraded while the other strand represents the mature miRNA, which is integrated into the RISC, acquiring the ability to repress translation (Figure 3) [30].

The incorporation of miRNAs into the exosomes can be exosome type-specific, in which case processes such as the neural sphingomyelinase 2-dependent pathway [31] and the miRNA-induced silencing complex-related pathway regulate the sorting of miRNAs [32]. It can also be independent of the miRNA sequence, being mainly controlled by enzymes and other function-related proteins [33].

Taking into account all miRNA subcategories, a very broad class is represented by neuronal miRNAs, which comprise about 70% of all miRNAs in our body [34]. Neuronal miRNAs have very important functions in the CNS, among which are neuronal development, neurogenesis, and synaptic plasticity. A single neuronal miRNA molecule can interact with hundreds of other mRNA molecules, even without full complementarity [35,36]. As a result, neuronal miRNAs are integral regulatory molecules in both physiological conditions of the CNS and in disease states, which makes them an important research area to focus on.

## 2. The Role of Exosomes and Exosomal miRNAs in the CNS

In the brain, exosomes are involved in various, often overlapping processes. Such processes include cell-to-cell communication, neurogenesis, synaptic plasticity, and stress response.

### 2.1. Cell-to-Cell Communication

Neurons contain a high number of MVBs in their soma and dendrites but not along their axons [37]. This observation led to the hypothesis that exosomes traveling along the neuraxes have a specific role in the neural synapses, taking thus part in cell-to-cell communication. Indeed, exosomes are critical for signal transmission in the CNS as they act as vehicles of communication between cells of the same or different types, particularly between neurons and glia. The release of exosomes from CNS cells is a process controlled by synaptic glutamatergic activity and calcium influx [38,39].

### 2.2. Neurogenesis

Exosomes may both promote and hinder adult hippocampal neurogenesis (AHN), as shown by Luarte et al. [40]. Furthermore, the injection of cultured exosomes containing known pathogens into the dentate gyrus in mouse hippocampi caused the significant impairment of AHN [41].

Of all miRNAs, the most prevalent in the brain is miR-124 since it is a key regulator of adult neurogenesis by targeting cAMP response element-binding protein (CREB) [42,43]. If dysregulated, miR-124 can cause significant neurodegeneration. Also involved in neurogenesis are miR-137, which regulates the differentiation of neural stem cells into mature neurons [44], and miR-125b, which mediates axon outgrowth [45,46].

### 2.3. Synaptic Plasticity

Synaptic plasticity is of great importance because it is involved in the processes of learning and memory, and if disrupted, can cause a range of psychiatric disorders. As revealed by the protein analysis of exosomes in the CNS, it is greatly influenced by exosomes [47,48]. Goldie et al. showed that neuron-to-glia signaling via exosomes helps active synapses stimulate the pruning of neighboring synapses that are inactive. More specifically, they identified microtubule-associated protein 1B (MAP1b), a protein essential to synaptic plasticity, in exosomes from depolarized human neurons in culture [49]. A deficit in the synaptic signal transduction and over-abundance of cortical miRNAs in patients with schizophrenia suggested that miRNAs are important components in the regulation of neuronal plasticity [50,51]. Furthermore, Bahrini et al. showed that when microglial cells were incubated with neuron-derived exosomes, there was an increase in the expression of complement component 3 (C3) in the microglial cells, which facilitated the removal of neurites [52].

Both Schratt et al. and Le et al. suggested that miR-132, -134, and—let-7 were important in regulating synaptic plasticity [45,46]. Castrén and Rantamäki showed that several miRNAs influenced the expression of the gene encoding brain-derived neurotrophic factor (BDNF), which in turn promoted the synthesis of miR-132, increasing neurogenesis [53]. They proposed that BDNF is involved in synaptic plasticity and that this is important for the recovery from depression.

### 2.4. Stress Response

CNS signaling via exosomes is involved in important processes, including the stress response. Indeed, neurons release various neurotransmitters, which stimulate the secretion of exosomes from the oligodendrocytes. In turn, neurons internalize these oligodendrocyte-derived exosomes, which offer them cellular protection, as their cargo increases the tolerance of neurons to stress [54].

Neurons are also protected from stress via astrocyte-derived exosomes through the prion protein (PrP) and other neuroprotective molecules. According to Guitart et al., the PrP contained in astrocyte-derived exosomes protects neurons from stress conditions like ischemia, hypoxia, hypoglycemia, and oxidative stress [55]. Apart from PrP, astrocyte-derived exosomes contain synapsin 1, heat shock protein 70, and matrix metalloproteinases [56,57]. As a result, upon heat, hypoxia, or any other stressful stimulus, astrocyte exosomes increase the viability of neural cells [58].

### 2.5. Mitochondrial Function in Brain

The mitochondria play a pivotal role in brain activity; therefore, in recent years, escalating research has focused on the profound impact of mitochondrial dysfunction on the pathogenesis of mental health conditions. Beyond their role as the primary energy source of the cell, mitochondria oversee many processes vital for cellular survival, including redox signaling and calcium transportation. This intricate web of functions underscores the indispensability of mitochondria in various network processes, emphasizing their central role in CNS homeostasis and pathological states. The “mitochondrial hypothesis” posits that mitochondrial dysfunction is intricately linked to a broad spectrum of processes influencing the progression and severity of mental disorders [59].

Impaired mitochondrial functioning may result from several causes, including alterations in mitochondrial gene expression. In that respect, a subcategory of miRNAs is called ‘mitochondrial miRNAs’ (mitomiRs). Named after their site of action, mitomiRs can originate from the nucleic DNA and then be transported into the mitochondria or can be transcribed directly from the mitochondrial DNA. The highly coiled, circular human mitochondrial DNA houses 37 genes and approximately 150 miRNA sequences [60]. However, due to the absence of the Dicer and Drosha enzymes, the transcription and biogenesis of mitomiRs in mitochondria remain largely unknown [61]. There is evidence that mitomiRs are involved in several critical mitochondrial functions such as oxidative phosphorylation (OXPHOS), electron transport chain (ETC) components, redox signaling, lipid metabolism, mitochondrial membrane potential, and the transportation of calcium [62]. In addition, mitomiRs may influence nuclear gene expression and mRNA processing through mitochondrial retrograde signaling in the host cell or through their incorporation in exosomes in other cells [63].

## 3. Stress-Induced Changes in CNS Exosomal Functions

According to Chrousos, “All organisms must preserve a complex dynamic equilibrium, or homeostasis, which is repeatedly challenged by internal or external adverse forces termed stressors. Stress occurs when homeostasis is threatened or perceived to be so; homeostasis is re-established by various physiological and behavioral adaptive changes” [64].

Although stress forces virtually all biological systems away from a physiological steady state, it is a fundamental element of the natural world that may lead to adaptational changes and progressive individual and species improvement. Thus, in acute, transient, motivating, and surmountable stress states, adaptive responses are beneficial. In contrast, in prolonged stress, when the adjustive capacity of the organism is exceeded, the adaptive changes may become detrimental, damaging cerebral functions and favoring the development of mental diseases [64].

### 3.1. Neurogenesis

As reported by Luarte et al., miRNAs derived from astrocytes possibly regulate neurogenesis under stress. In fact, astrocyte-derived miRNAs normally take part in neurogenic processes and are modulated by different stimuli. Under stress conditions, they may be up- or down-regulated and secreted via exosomes into the interstitial glymphatic space [65], contributing to cellular communication between astrocytes and neurons [40].

Focusing on microglia, Fan et al. showed that miR-146a-5p hinders neurogenesis by targeting Krüppel-like factor 4 (KLF4) [66]. Furthermore, Wei et al. demonstrated both in vivo and in vitro that miR-139-5p is a negative regulator of neural stem cell proliferation and differentiation. Moreover, when they isolated blood exosomes from major depressive disorder (MDD) patients and injected them peripherally into mice, they observed the appearance of depressive-like behaviors in the experimental animals [67].

### 3.2. Neuroinflammation

There is extensive evidence for the roles of exosomes in stress-induced neuroinflammation. First, Frühbeis et al. showed that reactive microglia released exosomes and microvesicles (MVs) carrying the pro-inflammatory cytokine interleukin-1β (IL-1β), the IL-1β processing enzyme caspase-1, and the purinergic receptor P2X 7 (P2RX7), all of which may induce and propagate inflammatory reactions throughout the brain [68]. Moreover, according to Dozio and Sanchez, the exosomal protein cargo from brain endothelial cells is modified after exposure to the pro-inflammatory cytokine tumor necrosis factor alpha (TNF-a) [69]. They carry proteins involved in the signaling pathways of TNF-a and nuclear factor kappa-light-chain-enhancer in activated B cells (NF-κB), which may cause the low-level, chronic neuroinflammation associated with depression [70].

In addition, monocytes activated by interferon alpha (IFNα) release exosomes with altered miRNA profiles that could influence brain macrovascular endothelial cells (BMECs) and trigger an inflammatory reaction [71]. Taken into consideration, along with analyses of blood–brain barrier (BBB) permeability in mental disorders, the above evidence suggests that changes in BMECs could result in a leaky BBB, extended neuroinflammation, and, eventually, in the onset or progression of mental disorders [72].

Another miRNA, miR-9-5p, can cause significant neuronal injury. In a study, when this miRNA was transferred as an exosomal cargo from neurons to microglia, it led to the polarization of the latter into the M1 type, damaging brain tissue [73].

## 4. The Role of Exosomes in the Pathogenesis of Mental Disorders

Mental disorders comprise major causes of misery and disability in humans, who have been suffering for many centuries from prejudices and social exclusion, with the exception of scientific humanistic approach [74]. Their prevalence has been increasing significantly during the last few years, especially during and after the COVID-19 pandemic. Recent research in the field of exosomes and exosomal miRNAs allows some insight into CNS pathophysiology. To our knowledge, exosomes have been proven to take part in the development of neuropsychiatric disorders mainly through their cargo, exosomal miRNAs, as the experimental evidence linking exosomes with mental disorders is very limited. Several exosomal cargo molecules (especially miRNAs) have been deeply investigated for their roles in the pathophysiology of mental diseases. Therefore, in our review, we mostly include papers evaluating the role of exosomal mediators (miRNAs) and not of exosomes. This does not exclude the fact that exosomes could also be involved but, again, the experimental evidence is very limited. In addition, although not directly related to exosomes, the involvement of mitomiRs in neuropsychiatric disorders is also discussed because we believe that mitomiRs constitute a very important field with great potential in epigenetics, which has not been thoroughly studied yet. In the following sections, we discuss the possible roles of exosomal miRNAs in the pathophysiology of common neuropsychiatric disorders, namely schizophrenia, major depression, bipolar disorder, and Alzheimer’s and Huntington’s’ diseases (summarized in Table 1).

### 4.1. Schizophrenia

Schizophrenia (SCZ) is a chronic mental disorder that is characterized by motivational, cognitive, and affective dysfunction and psychosis. At an international level, one in two-hundred-and-twenty-two adults (0.45%) is affected by schizophrenia and has a two- to three-times-increased risk of dying earlier than the general population [98]. The pathogenesis of SCZ has a diverse genetic, epigenetic, and neurobiological basis, which involves alterations in brain structure and function, where dopamine dysregulation is shown to play a central role [99].

Exosomes may be involved in the pathophysiology of schizophrenia (SCZ) in multiple ways, mainly by causing neurotoxicity. Glial cells secrete, through exosomes, N-methyl D-aspartate receptor C (NMDAR-C) C-C motif chemokine receptor 2 (CCR2), NMDAR-C-X-C chemokine receptor type 4 (CXCR4), and NMDAR- interleukin 1 receptor type II (IL1R2) heteromers, which may form complexes with NMDARs, reducing NMDAR signaling [100]. In addition, analysis of the blood exosomes of SCZ patients has shown the up-regulation of exosomal miR-206, which suppresses the expression of BDNF mRNA and protein [97].

Furthermore, mitochondrial dysfunction and oxidative stress contribute to SCZ pathogenesis [101,102,103,104]. In the blood of early psychosis patients, oxidative stress causes the up-regulation of the miR-137, resulting in decreased cytochrome c oxidase subunit 6A2 (COX6A2) and the accumulation of impaired mitochondria [96]. This mitochondrial dysfunction worsens the oxidative stress levels in the cell and damages parvalbumin interneurons. The impairment of mitochondria is also correlated with social behavioral impairment via the sequestration of gamma-aminobutyric acid (GABA) and lower GABAergic signaling [104].

Tan et al. recently showed that the expression of four exosomal circular RNAs (circRNAs) is modified in the plasma of SCZ patients. These circRNAs contain binding sites for specific miRNAs and act as sponges that bind to miRNAs and impede the miRNA-induced gene silencing. The circular RNAs that were found to be altered in SCZ targeted miR-34a-5p and miR-499a, which have been proven to take part in the pathophysiology of SCZ. These circRNAs are associated with SCZ also by taking part in histone ubiquitination, metabolic activities, and stress reaction. Other circRNAs are also enriched in signaling pathways crucial for adult neurogenesis such as the Notch and Mitogen-activated protein kinase (MAPK) pathways [95,105,106].

### 4.2. Major Depressive Disorder

MDD is a mood disorder characterized by feelings of sadness, emptiness, or irritable mood, as well as by cognitive and somatic changes. It affects more than 264 million people of all ages worldwide [107]. Although extensively studied, a large part of the pathophysiology of depression remains elusive. There is a need to identify new research targets, such as exosomes and exosomal miRNAs, whose levels are greatly dysregulated in depressed patients.

In depression, exosomes containing miRNAs target the serotoninergic system (SERT, 5HT1A, 5HT1D) while there is a consistent alteration in the levels of miRNAs that regulate the MAPK and Wingless-related integration site (Wnt) pathways. Some miRNAs are shared with SCZ and target BDNF, Ataxin 1 (ATXN1), the glutamatergic system, and other signaling pathways [108]. miRNAs unique for depression pathophysiology are miR-451a, miR-1202, miR-135a, miR-425-3p, miR-34a-5p, miR-335-5p, miR-26a, miR-24-3p, miR-221-3p, and let-7d [88,89,90,91,93,94,109,110]. The levels of all the miRNAs mentioned above have been investigated by detecting the exosomes in the peripheral blood of human patients with the aim of highlighting a miRNA as a potential biomarker for MDD.

Interestingly, mitomiRs are also involved in the pathophysiology of MDD. It is known that in states of cellular stress such as inflammation, mitochondrial genes are fragmented and released to the peripheral blood, resulting in circulating cell-free mitochondrial DNA (ccf-mtDNA) [111]. ccf-mtDNA acts as a damage-associated molecular pattern (DAMP) in the plasma and triggers innate immunity [112]. Indeed, in a study involving untreated MDD patients, there was a positive correlation between the expression levels of five plasma miRNAs (hsa-miR-6068, hsa-miR-939-5p, hsa-miR-187-5p, hsa-miR7110-5p, and hsa-miR-4707-3p) with the plasma ccf-mtDNA copy number, indicating a common mechanism of action [92]. These mitomiRs are hypothesized to elevate reactive oxygen species (ROS) and produce ccf-mtDNA by impairing cytochrome oxidase complexes I and III. However, additional research is required to confirm it.

### 4.3. Bipolar Disorder

Bipolar disorder (BD) is a chronic mood disorder in which affected patients experience alternating depressive and manic episodes with intermittent periods of mood stability (euthymia). It is calculated that 4.4% of adults in the United States are affected by bipolar disorder at least once in their lives [113]. Recent evidence indicates that dysregulated miRNAs in BD target pathways such as neural signaling and synaptic plasticity, and there is promising evidence that exosomes, which primarily function as miRNA carriers, play a key role in the pathogenesis of BD. This has been confirmed both by the observation that miRNA levels are altered in patients and by genetic research that has found MIR genes within loci of susceptibility for BD [85].

In their review, Fries et al. suggest that the most promising miRNAs in BD are miR-34a and miR-137 [83,84,85,86]. In the analysis of exosomal miRNAs in the postmortem brain tissue of BD patients, miR-34a levels were increased in the cerebellum and decreased in the anterior cingulate cortex [83,84]. miR-34a levels were also found to be altered in cultures of lymphoblastoid cells of BD patients; 7 and 16 days after the cells were treated with lithium, miR-34a levels were increased [114].

Regarding miR137, a risk allele of a rare enhancer single nucleotide polymorphism (SNP) (1:g.98515539A > T) has been described to affect the miR137 gene. The levels of miR-137 are specifically decreased by two functional variants (rs185304769 and rs1198588). As a result, physiological mechanisms are possibly hindered, and pathologic changes associated with neuropsychiatric diseases are induced [115]. Duan et al. proved this by showing that this risk allele was more frequently found in BD than healthy patients [116].

Ceylan et al., who performed the miRNA sequencing of plasma exosomes from BD patients and healthy individuals, identified 13 aberrant miRNAs. Among them, the level of miR-185-5p was significantly increased while the levels of miR-484, miR-652-3p, and miR142-3p were decreased [87]. miR-484 takes part in regulatory pathways of neurogenesis, the mitochondrial network, and redox modulations, processes that are closely related to the pathophysiology of BD [117,118]. Furthermore, miR-142-3p has an important signaling function during embryonic development, homeostasis, and disease. This miRNA, which has been shown to be dysregulated in exosomal samples of patients with BD, had previously been shown to be down-regulated in unipolar depression patients [119,120,121]. Another down-regulated miRNA in patients with BD was miR652-3p, which is involved in immune processes and oxidative stress mechanisms [108,122]. In addition, miR185-5p, the only up-regulated miRNA, has a possible involvement in the regulation of tyrosine kinase receptor type 2 [123], which has been related to anxiety, depression, and suicide [124].

### 4.4. Alzheimer’s Disease

Alzheimer’s disease (AD), the most common type of dementia, is a neurodegenerative disorder characterized by progressive cognitive and functional decline related to age and accompanied by a unique neuropathology. It accounts for 50–70% of dementia cases [125] and affects 10–30% of the population aged 65 years and older [126]. The deposition of hyperphosphorylated Tau protein and APP fragment beta-amyloid (Aβ) results in synaptic loss and neuronal atrophy, mainly in the hippocampus and the cerebral cortex [127]. Except for memory loss and impairment of executive functioning, AD involves a range of neuropsychiatric symptoms like apathy, disinhibition, agitation, and psychosis, which significantly impair behavior and mental health.

The role of exosomes in AD is dual, as they can have both neurotoxic and neuroprotective effects. On the one hand, exosomes induce the apoptosis of neurons as they take part in the secretion, transfer, and propagation of toxic Aβ and tau proteins, which are taken up by microglia and neurons. In this concept, Gao et al. showed that there is an increased expression of glutaminase C (GAC) in the microglia of AD mouse models. GAC activates microglia by enhancing the secretion of exosomes and by changing the exosomal content to contain more pro-inflammatory miRNAs [128].

On the other hand, the neural stem cells of the hippocampus produce exosomes that protect synapses against Aβ oligomer toxicity as they absorb Aβ and transport neuroprotective substances between cells [129,130,131]. In the work of Losurdo et al., mesenchymal stem cell (MSC)-derived exosomes induced an anti-inflammatory response in murine primary microglia in vitro. In addition, intranasally administered MSC-derived exosomes were delivered to the brain in mouse models, where they enhanced the density of dendritic spines and down-regulated the activity of microglial cells. By administering MSC-derived exosomes in a noninvasive manner, Losurdo et al. showed that the inhibition of inflammatory processes can have neuroprotective effects against AD [129].

Impaired synaptic activity is one of the hallmarks of AD, and mitomiRs have a central role in neurotransmission by modulating the generation of ATP and the buffering of calcium ions in mitochondria [132].

A study that investigated the expression of different miRNAs in the cerebrospinal fluid (CSF), brain tissue, and plasma of AD patients detected increased levels of miR-15a in all three tissue types. The functions of miR-15a are important for late-onset AD. miR-15a regulates the expression of CD2-associated protein, which takes part in the preservation of BBB integrity [133]. Specifically related to mitochondria, miR-15a/b targets the mRNA of the β-site amyloid precursor protein cleaving enzyme 1 (BACE1) [75], which creates neurotoxic β-amyloid plaques by cleaving the Aβ precursor protein [134].

Another study by Russell et al. demonstrated the involvement of miR-34 in a TNF-a-induced pathological pathway in Alzheimer’s disease. TNF-a-stimulated miR-34a silences the mRNA expression of five key proteins in the mitochondrial electron transport chain (Complex I: NDUFC2, Complex II: SDHC, Complex III: UQCRB and UQCRQ, and Complex IV: COX10). This impairs mitochondrial function, causing the cellular accumulation of APP and Aβ42. Moreover, the oligomeric amyloid beta 42 (oAβ42) stimulates miR-34a, leading to a vicious cycle that exacerbates the neuropathology of AD [77].

The above examples are indicative of the critical role that mitomiRs play in regulating mitochondrial function under the pathological conditions of AD. There are currently a lot of reporting miRNAs that regulate mitochondrial functions in AD, and a synopsis of them was described by John et al. in their review [135].

### 4.5. Huntington’s Disease

Huntington’s disease (HD) is an autosomal dominant neurodegenerative disorder that affects patients in the fourth to sixth decades of their lives and is characterized by involuntary chorea-like movements, along with cognitive and behavioral impairment. The prevalence of HD at an international level is calculated to be 4.88 per 100,000 persons [136]. In HD, there is a loss of medium spiny neurons in the CNS caused by the misfolding and successive intracellular accumulation of a mutant form of huntingtin (HTT) [137]. The huntingtin gene, located in the short arm of chromosome 4p16.3, contains a polymorphic CAG repeat, which becomes pathological if it comprises more than 35 CAG trinucleotide repeats [138]. The mutant range CAG repeats result in a polyglutamine tract in huntingtin, which is responsible for the toxicity of the protein [138]. The more the CAG repeats, the earlier the disease onset will be.

Exosomes mediate the progression of neurodegenerative diseases by integrating pathogenic proteins and RNAs into ILVs and releasing them into the extracellular environment [139]. In HD, the pathogenic protein is mutant huntingtin (mHTT), which is spread through cells via exosomes. This was shown by animal experiments that demonstrated the transmission of mHTT aggregates between cells [138,140] and by experiments of cytoplasmic aggregate formation [141].

Some miRNAs are commonly associated with HD.

miR-9 was found to be down-regulated in the brain in HD patients. miR-9 participates in transcriptional dysregulation by targeting two components of the RE1-silencing transcription factor (REST), which inactivates neuron-specific genes in HD [79].

MiR-10b-5p was found to be up-regulated in the prefrontal cortex in HD patients. This miRNA down-regulates the mRNA of the mHTT protein and inhibits the ex-pression of the BDNF gene. It is not clear whether this has a neuroprotective effect or not [80].

MiR-196a was found to be associated with mHTT expression as high miR-196a levels suppressed the expression of mHTT in the brain in HD mouse models, cell cultures, and induced pluripotent stem cell models. Cheng et al. hypothesize that miR-196a takes part in several neuronal regulatory pathways such as the CREB protein pathway and ubiquitination pathways [82].

Some mitomiRs may also be involved in HD by modulating mitochondrial dynamics, biogenesis, and mitophagy.

After an induction of miR-214 expression in cell cultures, miR-214 was associated with suppressed Mitofusin 2 (MFN2) [83]. MFN2 takes part in mitochondrial fusion–fission and the miR-214-induced decrease in MFN levels results in fragmented mitochondria [142]. Mitochondrial impairment caused by abnormal fusion-fission cycles has been correlated with many neuropsychiatric diseases, including HD [143].

MiR-218 is another miRNA that was found to be associated with mitochondrial dysfunction and HD. miR-218 interacts with Parkin RBR E3 ubiquitin ligase (PRKN), a crucial enzyme for the PINK1/PRKN-mediated mitophagy pathway [81]. When miR-218 is over-expressed in cell cultures, miR-218 down-regulates the expression of PRKN, hindering the normal mitophagy process. The problematic mitochondrial clearance disrupts cellular homeostasis and is correlated with the cellular dysfunctions described in HD [144].

### 4.6. Critical View of Neuropsychiatric Diseases That Share Similarities in Alterations of miRNA Levels

In MDD, miRNAs, such as miR-146b-5p, involved in different MAPK and Wnt signaling pathways, have been observed to be consistently altered. Interestingly, the same miRNAs are altered in BD [108]. It is known that the MAPK pathway is involved in neurological processes like the initiation of cortical neurogenesis and plasticity while the Wnt pathway plays a crucial role in diverse stages of neurogenesis during early development and adulthood. The alteration of the levels of the same miRNAs for these pathways in MDD and BD confirms our knowledge that neurogenesis and plasticity are directly related to both of these mood disorders [108].

In both MDD and SCZ, there are alterations in the levels of the same miRNAs that target BDNF signaling pathways. The role of BDNF in the pathogenesis of MDD has been well established as it is associated with maladaptive neuroplasticity [145]. Meanwhile, human and animal studies have shown that BDNF is related to SCZ-like phenotypes and that dysregulated BDNF signaling causes alterations in neurodevelopment. However, although a subthreshold association exists, the BDNF locus has not been officially correlated with SCZ through genome-wide association studies. A better comprehension of BDNF signaling during development will possibly explain the role of this gene in SCZ.

Furthermore, miR-146a-5pa is up-regulated in both AD and HD. It has been proposed that in AD, miR-146a-5pa activates MAPK signaling, triggers oxidative stress, and, via this mechanism, increases Aβ deposition [78]. miR-146a also targets human and mouse HTT genes, while decreased miR-146a expression increases the expression of Checkpoint Kinase 1 (CHEK1) and Cyclin A2 (CCNA2), which rescue the cell cycle. In addition, dysregulated miR-146a-5pa may contribute to HD pathogenesis by targeting TATA-binding protein (TBP) [146] miR-146a-5pa may also be involved in several other pathways possibly common for AD and HD, a potentiality that might explain the amnesia-like and neuropsychiatric manifestations that appear in both of these CNS diseases.

## 5. Conclusions

While clinical research was classically focused on genomics and proteomics, more and more attention is currently being given to the field of epigenomics. The study of epigenetic changes in cells has revealed the remarkable properties of exosomes and exosomal miRNAs and their potential to function as diagnostic and therapeutic molecules. Especially for neuropsychiatric diseases, CNS-derived exosomes have immense biomarker and therapeutic potential.

Regarding their biomarker potential, exosomes and exosomal mRNAs are appealing targets for a lot of reasons. Firstly, the cargo of exosomes is modified in pathological conditions, with exosomal miRNAs mirroring the disease presence [147]. In addition, because of the highly stable exosomal membrane, the contents of exosomes can be preserved for long periods of time before sample analysis. Specifically for CNS-derived exosomes, their ability to cross the BBB makes it feasible to isolate them noninvasively from peripheral biological fluids such as urine, blood, and saliva. Of course, exosomes can be traced to their cells of origin by utilizing CNS-specific biomarker molecules on the exosomal surface. For example, exosomes that are secreted by microglia, oligodendrocytes, and neurons have, on their surfaces, antibodies against CD13, proteolipid protein (PLP), and L1 cell adhesion molecule (L1CAM), respectively [148,149]. The easy detection of CNS-derived exosomes allows for a timely diagnosis of neuropsychiatric disorders, increasing the chances for a better outcome.

The ability to cross the BBB makes it possible not only to detect CNS-derived exosomes in the periphery but also to deliver peripherally inserted exosomes in the CNS, utilizing them as drug carriers. The content of the exosomal drug carriers could be miRNA, whose therapeutic effects have long been proved in clinical settings to a wide variety of molecules such as various metabolites, proteins, and nucleic acids. The low immunogenicity and the strong biodegradability of exosomes would optimize drug content delivery to the CNS and would reduce the adverse effects [150].

Bringing exosomes and exosomal miRNAs from the bench to the bedside has great potential but there are still a lot of unanswered questions and technical limitations that need to be surpassed. There is still a need for a better distinction of exosomal particles from other kinds of particles. For better exosome separation and quantification, there is a need for new and more efficient isolation techniques. Studies have shown that the quality and purity of the isolated exosomes can be important for the isolation technique that is used [151]. In our review, we have mentioned the clinical studies where miRNA measurements come from purified exosomes, but this is not generally the case as total blood is used more commonly for miRNA isolation. Moreover, there are still gaps in our knowledge about the secretion mechanisms of exosomes. Although there has been significant progress in the identification of biomarker surface molecules, it is still often challenging to confirm where blood exosomes come from. Finally, many functions of the exosomes need to be researched in more depth. For example, it is still questionable whether the main function of exosomes in the brain is to mediate cell-to-cell communication or to discard toxic mediators. This and a lot of more unclear points must be answered before the exosomes can be clinically applied.

In conclusion, the following can be stated:

(a) Exosomes constitute an evolutionarily conserved, universal mechanism of communication between cells;
(b) Exosomal miRNAs regulate a wide range of biological processes by binding to complementary sequences and modulating gene expression and mRNA translation;
(c) Exosomal miRNAs control the response to stress in neurons and glial cells by modifying their morphology and function in response to different stimuli [6,18];
(d) Exosomal miRNAs have been associated with neurodegeneration and psychopathology. They have crucial regulatory roles in the interface between genetic information, external (environmental) stimuli, and brain structure and function;
(e) The current knowledge of the exosomal ability to transfer genetic information between cells is cultivating a fertile ground for the design of therapeutic interventions to alleviate disease;
(f) New discoveries regarding the role of miRNAs in the CNS promise to be unpredictable and astonishing, while they could help decode the very essence of human life.

## Figures and Tables

**Table 1:** MicroRNA (miRNA) that have been documented to exhibit increased (↑) or decreased (↓) expression levels in Alzheimer’s disease (AD), Huntington’s disease (HD), bipolar disorder (BD), major depressive disorder (MDD), and schizophrenia (SCZ).

Disease	miRNA	Alteration	Model	Tissue Type	Significance	Refs.
Alzheimer’s disease	miR-15a	↑	Human patients	CSF	Causes mitochondrial dysfunction and unbalances mitochondrial membrane potential	[75,76]
	miR-34	↓	Human patients and AD mouse models	Brain tissue	Down-regulates five key proteins in the mitochondrial electron transport chain	[77]
	miR-146a-5pa	↑	Mouse models	Brain tissue; hippocampus	Triggers oxidative stress through MAPK signaling	[78]
Huntington’s disease	miR-9	↓	Human patients	Brain tissue; Brodmann’s area 4 (BA4) cortex	Targets two components of the REST (RE1-silencing transcription factor), which inactivates neuron-specific genes	[79]
	miR-10b-5p	↑	Human patients	Brain tissue; Prefrontal cortex	Targets HTT and reduces its expression. May down-regulate the translation of BDNF	[80]
	miR-218	↑	Cell cultures		Targets PRKN E3 ubiquitin ligase and inhibits mitochondrial clearance	[81]
	miR-196a	↑	Mouse models and cell cultures	Brain tissue	Controls the expression of CBP and PGC-1α in HD	[82]
	miR-214	↑	Cell cultures		Down-regulates the expression of MFN2, aggravating the distribution of fragmented mitochondria	[83]
Bipolar disorder	miR-34a	↑ [84]/↓ [85]	Human patients [84,85]	Postmortem brain tissue; cerebellar [84]/anterior cingulate cortex [85]	Targets NCOA1, altering glucocorticoid receptor’s transcriptional activity	[84,85]
	miR-137	↑	Human patients	Peripheral blood	Disruption of its expression deregulates neuronal proliferation and differentiation	[86]
	miR-142-3p	↓	Human patients	Peripheral blood	Takes part in signaling pathways during embryonic development	[87]
	miR-185-5p	↑	Human patients	Peripheral blood	Takes part in the regulation of tyrosine kinase receptor type 2, which has been associated with anxiety, depression, and suicide	[87]
	miR-484	↓	Human patients	Peripheral blood	Takes part in regulatory pathways of neurogenesis	[87]
	miR-652-3p	↓	Human patients	Peripheral blood	Takes part in immune processes and oxidative stress mechanisms	[87]
Major depressive disorder	let-7d	↑	Human patients	CSF and peripheral blood	Possible biomarker for MDD	[88]
	miR-24-3p	↑	Human patients and mouse models	Peripheral blood, postmortem brain tissue	Possible marker of treatment response and regulator of the MAPK/Wnt systems	[89]
	miR-26a	↑	Human patients	Peripheral blood	Possible marker of treatment response	[90]
	miR-34a-5p	↑	Human patients	CSF and peripheral blood	Possible biomarker for MDD	[88]
	miR-135a	↓	Human patients	Peripheral blood and brain tissue	Controls 5HT levels and is a possible biomarker for MDD	[91]
	miR-187-5p	-	Human patients	Peripheral blood	Positive correlation with ccf-mtDNA copy number	[92]
	miR-221-3p	↑	Human patients	CSF and peripheral blood	Possible biomarker for MDD	[88]
	miR-320a	↓	Human patients	Peripheral blood	Targets GRIN2A and DISC1	[93]
	miR-425-3p	↑	Human patients and mouse models	Peripheral blood	Possible marker of treatment response and regulator of the MAPK/Wnt systems	[89]
	miR-451a	↑	Human patients	Peripheral blood	Levels related to ketamine treatment. Associated with SLC17A7, which is significantly decreased in depression	[93]
	miR-939-5p	-	Human patients	Peripheral blood	Positive correlation with ccf-mtDNA copy number	[92]
	miR-1202	↓	Human patients	Peripheral blood	Possible biomarker for clinical response to antidepressant monotherapy	[94]
	miR-4707-3p	-	Human patients	Peripheral blood	Positive correlation with ccf-mtDNA copy number	[92]
	miR-6068	-	Human patients	Peripheral blood	Positive correlation with ccf-mtDNA copy number	[92]
	miR-7110-5p	-	Human patients	Peripheral blood	Positive correlation with ccf-mtDNA copy number	[92]
Schizophrenia	miR-34a-5p + miR-499a	↑	Human patients	Peripheral blood	Have potential as biomarkers for schizophrenia	[95]
	miR-137	↑	Human patients and mouse models	Peripheral blood and brain tissue; prefrontal cortex	Damages mitochondria, causing oxidative stress and parvalbumin interneuron impairment.	[96]
	miR-206	↑	Human patients	Peripheral blood	Regulates BDNF expression	[97]

## Data Availability

No new data were created or analyzed in this study.

## Abbreviations

AD	Alzheimer’s disease
AHN	adult hippocampal neurogenesis
Alix protein	ALG2-interacting protein X
ATXN1	Ataxin 1
Aβ	beta-amyloid
BACE1	β-site APP-cleaving enzyme 1
BBB	blood–brain barrier
BD	bipolar disorder
BDNF	brain-derived neurotrophic factor
BMECs	brain macrovascular endothelial cells
C3	complement component 3
ccf-mtDNA	circulating cell-free mitochondrial DNA
CCNA2	Cyclin A2
CCR2	C-C motif chemokine receptor 2
CHEK1	Checkpoint Kinase 1
circRNAs	circular RNAs
CNS	central nervous system
COX6A2	cytochrome c oxidase subunit 6A2
CREB	cAMP response element-binding protein
CSF	cerebrospinal fluid
CXCR4	C-X-C chemokine receptor type 4
DAMP	damage-associated molecular pattern
Drosha-DGCR8	DiGeorge syndrome Critical Region gene 8
ETC	electron transport chain
ESCRT	endosomal sorting complex required for transport
EV	extracellular vesicle
GABA	gamma-aminobutyric acid
GAC	glutaminase C
HD	Huntington’s disease
Hsp70	heat shock protein 70
HTT	Huntingtin
IFNα	interferon alpha
IL1R2	interleukin 1 receptor type II
IL-1β	interleukin-1β
ILV	intraluminal vesicle
KLF4	Krüppel-like factor 4
L1CAM	L1 cell adhesion molecule
lncRNA	long non-coding RNA
MAP1b	microtubule-associated protein 1B
MAPK pathway	Mitogen-activated protein kinase
MDD	major depressive disorder
MFN2	Mitofusin 2
mHTT	mutant Huntingtin
miRNA	microRNA
mitomiR	mitochondrial miRNA
MV	microvesicle
MVB	multivesicular body
ncRNA	non-coding RNA
NF-Κb	nuclear factor kappa-light-chain-enhancer of activated B cells
NMDAR-C	N-methyl D-aspartate receptor C
OXPHOS	oxidative phosphorylation
P2RX7	purinergic receptor P2X 7
PGC-1α	peroxisome proliferator-activated receptor-γ coactivator-1α
PLP	proteolipid protein
PRKN	Parkin RBR E3 ubiquitin ligase
PrP	prion protein
RISC	RNA-mediated silencing complex
ROS	reactive oxygen species
SCZ	schizophrenia
SNARE protein	SNAP receptor protein
SNP	single nucleotide polymorphism
TBP	TATA-binding protein
TNF-a	tumor necrosis factor alpha
TRBP	transactivation-responsive RNA binding protein
TSG101	tumor susceptibility gene 101 protein
UTR	untranslated region
Wnt pathway	Wingless-related integration site
